# Effect of submucosal cryotherapy compared with steroids and NSAIDs injections on Substance P and Interleukin 6 pulpal release in experimentally induced pulpal inflammation in rabbits

**DOI:** 10.1590/1678-7757-2024-0017

**Published:** 2024-05-17

**Authors:** Mai SHALABI, Abeer H MAHRAN, Tarek ELSEWIFY

**Affiliations:** 1 Ain Shams University Faculty of Dentistry Endodontic Department Cairo Egypt Ain Shams University, Faculty of Dentistry, Endodontic Department, Cairo, Egypt.; 2 Gulf Medical University College of Dentistry Restorative Dental Sciences Department Ajman UAE Gulf Medical University, College of Dentistry, Restorative Dental Sciences Department, Ajman, UAE.

**Keywords:** Analgesics, Animal model, Cryotherapy, Inflammation, Interleukin 6, Substance P

## Abstract

**Objective:**

To compare the effect of submucosal cryotherapy using cold saline to dexamethasone sodium phosphate and diclofenac sodium injections on substance P and interleukin 6 release in experimentally induced pulpal inflammation in rabbits’ molar teeth.

**Methodology:**

Fifteen rabbits were randomly classified into 3 groups according to the submucosal injection given: cold saline, dexamethasone sodium phosphate, and diclofenac sodium. A split-mouth design was adopted, the right mandibular molars were experimental, and the left molars served as the control without injections. Intentional pulp exposures were created and left for 6 hours to induce pulpitis. Pulpal tissue was extracted and examined for SP and IL-6 levels using ELISA. Within each group, the level of cytokines released was measured for both control and experimental groups for intragroup comparison to determine the effect of injection. The percentage reduction of each mediator was calculated compared with the control side for intergroup comparison then the correlation between SP and IL-6 levels was analyzed using Spearman’s rank order correlation coefficient. Statistical analysis was performed, and the significance level was set at p<0.05.

**Results:**

Submucosal cryotherapy, dexamethasone sodium phosphate, and diclofenac sodium significantly reduced SP and IL-6 pulpal release. Submucosal cryotherapy significantly reduced SP more than and IL-6 more than dexamethasone sodium phosphate and diclofenac sodium. Pulpal reduction of SP and IL-6 showed a strong positive significant correlation.

**Conclusions:**

Submucosal cryotherapy reduces the pulpal release of SP and IL-6 and could be tested as an alternative to premedication to potentiate the effect of anesthesia and control postoperative endodontic pain.

## Introduction

A major cause of odontogenic pain is inflammation of the pulp tissue due to various stimuli such as caries, trauma, or iatrogenic errors. This inflammation is a complex multistep process involving multiple mediators with different functions that regulate and modulate the tissue response as well as pain perception.^1^

Neuropeptides are a wide group of mediators responsible for homeostatic regulation and repair processes in both normal and inflamed pulp. One of the most important neuropeptides is substance P (SP).^2^ It is produced in trigeminal cell bodies and travels to nerve endings, mainly C-fibers in the pulp tissue to be stored and released later when stimulated.^3^ It can stimulate a variety of resident and circulating cells to express pro-inflammatory cytokines, increasing capillary permeability and blood flow thus raising the local pulpal pressure.^4^ Studies have shown a significant increase in pulpal SP levels in patients demonstrating signs of irreversible pulpitis as well as in case of pulp irritation, such as deep cavity preparation or increased chemical and/or thermal stimulation.^5^ The use of antagonist for blocking SP receptors was also demonstrably effective in pain relief clinically, therefore, proving the hypothesis that suggests the contribution of SP in pain production.^6^

In the case of pulpitis, many pro-inflammatory cytokines are released, including interleukin 6 (IL6), by innate immunity cells.^7^ The IL6 levels increase in case of pulpal and periradicular inflammation compared with a normal healthy pulp tissue, thus supporting the hypothesis suggesting the local release of IL6 in endodontic inflammatory lesions. Intrathecal injection of anti-IL6 neutralizing antibody relieved those pain-related behaviors.^8^ Previous studies also demonstrated that IL6 plays a role in nociceptive and central sensitization.^9^

Studies have shown that obtaining proper anesthesia in cases diagnosed with irreversible pulpitis is significantly more challenging than in cases with normal healthy pulps.^10^ This challenge is attributed to the inflammatory changes and cytokines production such as IL1, 2, 6, 8, tumor necrosis factor alpha, as well as the high prostaglandins production.^11^ The tetrodotoxin-resistant sodium ion channels also increase due to this inflammation, and this class is proven to be resistant to anesthetics.^12^ The altered resting potentials of nerves during inflammation and the reduction in their thresholds of excitation could also be a significant cause for the failure of anesthesia.^13^ Hence the use of preoperative anti-inflammatory drugs as an attempt to improve the success rate of anesthesia as well as reduce post-operative pain experience has been recorded in literature.

The non-steroidal anti-inflammatory drugs (NSAIDs), such as diclofenac sodium, produce their therapeutic action via inhibiting the cyclooxygenase 2 (COX 2) enzyme activity, highly expressed in irreversible pulpitis cases, thus, decreasing the level of prostaglandins (PGs) as well as proinflammatory cytokines production, which means improving the efficacy of local anesthetics and thus reducing postoperative endodontic pain.^14^

The literature shows that glucocorticosteroids, including dexamethasone sodium phosphate, can reduce the acute inflammatory process by decreasing vasodilation and migration of neutrophils, as well as suppressing arachidonic acid synthesis from cell membrane phospholipids, therefore inhibiting COX and lipoxygenase pathways activity with subsequent reduction in PGs and leukotrienes synthesis.^15^

In the endodontic field, numerous studies have analyzed the ability of cryotherapy to control the level of postoperative pain in patients diagnosed with irreversible pulpitis. It has also been postulated that cryotherapy might result in a local anti-inflammatory action in periapical tissues.^16^ The ability of cryotherapy to reduce postoperative pain by final irrigation with cold saline, and application of cold packs intraorally or extra-orally has been studied and documented; however, no study was found discussing the effect of cryotherapy by submucosal infiltration injection of cold saline into the tissues surrounding the affected tooth.

The effect of submucosal cryotherapy using cold saline injection in cases of symptomatic irreversible pulpitis on substance P and interleukin 6 has not been investigated before. Therefore, the null hypothesis tested is that submucosal cryotherapy, steroids, and NSAID injection show no difference in the effect on SP and IL6 levels in cases of symptomatic irreversible pulpitis.

## Methodology

### Ethical considerations

Ethical approval was obtained from the local institutional review board. The experiment was approved by the local “Institutional Animal Care and Use Committee” of the animal house facility; following the Animal Research Reporting of *In Vivo* Experiments (ARRIVE) guidelines and Preferred Reporting Items for Animal Studies in Endodontology (PRIASE) 2021 guidelines.

### Sample size calculation

Statistical sample size calculation was not applicable before starting the study. No previous studies could be found in the literature adopting the split-mouth design in addition to testing a novel approach, which is applying cryotherapy in submucosal injection rather than the intracanal route.

The study started with five rabbits per group, using 4 teeth in each rabbit; 2 experimental and 2 control, yielding 10 experimental samples and 10 control samples per group (n=10). After analysis of the results, the Cohen’s d effect size was calculated resulting in a sample size of 8 (n=8) per group.

### Sample selection

A total of 15 male white New Zealand rabbits weighing 3-3.5 kilograms kept in well-ventilated cages in the animal house facility with proper nutrition and health conditions were selected.

### Sample Classification

**Figure 1:**
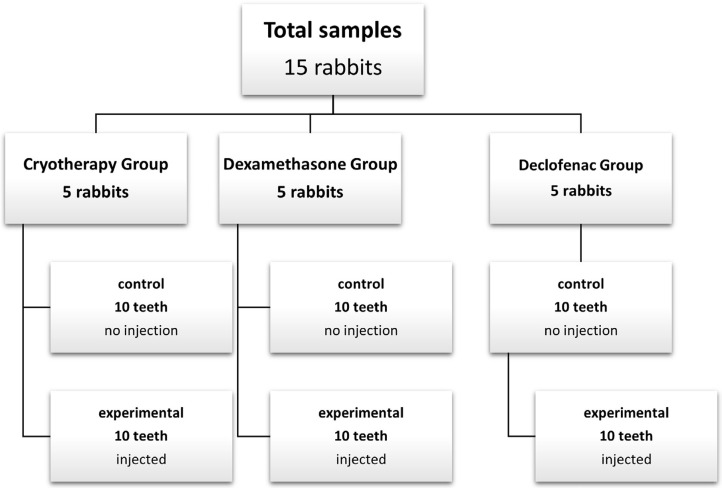
Diagram showing sample classification

Rabbits were equally and randomly divided into 3 groups according to the analgesic injected as shown in Figure 1 :

Cryotherapy Group: Submucosal injection of 1mL of 2 to 5°C cold saline.

Dexamethasone Group: Submucosal injection of 1ml dexamethasone sodium phosphate (4mg/ml)^17^

Diclofenac Group: Submucosal injection of 1 ml diclofenac sodium (25mg/ml)^18^

Within each group, control samples (in which exposure with no injection took place) were collected from the left mandibular molars, whereas experimental samples (in which injection was done before the exposure) were collected from the right mandibular molar teeth.

### Procedural steps

#### Anesthesia

Rabbits were anaesthetized by intraperitoneal injection of 3ml (20-40mg/kg) xylazine and 1.5ml (50mg/kg) ketamine.

#### Preparation and submucosal injection

Both cheeks of the rabbit were shaved using a safety blade razor and shaved hairs were cleaned away to avoid contamination. The cheeks were cut open using a number 22 surgical blade to allow better accessibility to the molars’ region.

Using a 1ml insulin syringe with attached 31-gauge needle, submucosal infiltration injection was performed at the first and at the third molar teeth. Injection was done in the depth of the vestibule of the right molar region, dividing the solution equally by injecting 0.5ml buccally and 0.5ml lingually at each molar.

#### Intentional exposure

**Figure 2:**
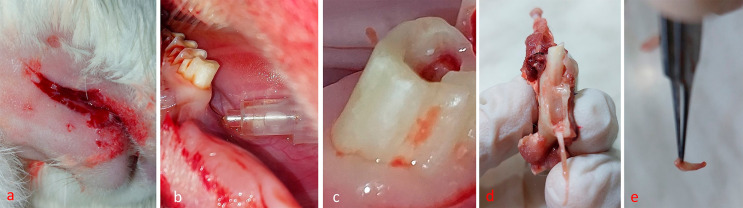
Steps of experimental procedure; a) cheeks shaved and surgically cut; b) infiltration injection; c) occlusal exposure in rabbit molar tooth; d) mandible cut open showing tooth in socket; e) pulpal tissue collection

Using an electric motor and a contra-angle hand piece operating at 5000rpm with a ¼ round bur, pulp was exposed by drilling through the center of the occlusal table of the rabbit’s first and third molar teeth, with simultaneous cooling with saline solution, until exposure is achieved. Rabbits’ cheeks were sutured using surgical suture until the time of euthanasia, 6 hours later. The exposed pulp was left open to the oral environment the entire time to induce an inflammatory reaction as shown in Figure 2 .^19^

#### Teeth extraction and pulp tissue sample collection

Rabbits were euthanized 6 hours after the procedure for teeth extraction and pulp sample collection.^19^

A straight hand piece with an attached disc was used to cut the mandibular bone for gentle extraction of the molar teeth, and the pulp tissue was collected from the root apices using a sterile H-file and ophthalmic forceps.

Excavated pulp tissue was immediately transported into Eppendorf tubes containing 1ml of sterile physiological saline solution and snap-frozen in liquid nitrogen to −80 degrees Celsius until examined using ELISA for the detection of SP and IL6.^20^ For each group the quantity of mediators produced in the pulp tissue were compared between the control and the experimental samples to determine the effect of injection of each solution and whether it reduced the expression of these mediators. The percentage reduction within each group was then calculated for intergroup comparison to determine which injection caused the greatest effect. A correlation between the reduction in SP and IL6 in the tested samples was calculated and statistically analyzed.

## Statistical analysis

Numerical data were presented as mean and standard deviation values. They were checked for normality using Shapiro-Wilk’s test. Data were normally distributed and analyzed using one-way ANOVA followed by Tukey’s *post hoc* test for intergroup comparisons and paired t-test for intragroup comparisons. Correlations were analyzed using Spearman’s rank-order correlation coefficient (rs). The significance level was set at p<0.05 in all tests.

## Results

### IL6

Table 1 shows the mean and standard deviation (SD) values calculated for the IL6 (pg/ml) produced in pulp tissue for intragroup comparison. All groups showed a significant reduction in the level of IL6 detected in pulp tissue (p<0.05).

**Table 1:** Mean±standard deviation of the amount of IL6 (pg/ml) produced in pulp tissue samples

Submucosal injection	IL6 (pg/mL) (mean±SD)		p-value	Cohen’s d
	Experimental	Control		
Cryotherapy	28.27±6.57	31.42±7.48	0.047*	−0.85
Corticosteroids	31.40±6.54	36.92±8.73	<0.001*	−1.94
NSAIDs	32.67±5.04	34.92±4.80	0.005*	−1.41

*: significant (p<0.05); ns: non-significant (p>0.05)

No statistically significant difference was found in the percentage reduction of IL6 between the 3 groups (p=0.383). The greatest percentage reduction in the level of IL6 was found with the cryotherapy group (13.55±3.99), followed by NSAIDs group (11.66±1.75), while the lowest percentage reduction was found with corticosteroids (11.53±3.40).

Table 1 : Mean±standard deviation of the amount of IL6 (pg/ml) produced in pulp tissue samples *.*

### SP

Table 2 shows the mean and standard deviation (SD) values calculated for the substance P (pg/ml) produced in pulp tissue for intragroup comparison. All groups showed SP levels in the experimental samples significantly reduced compared with the control samples (p<0.05).

**Table 2:** Mean±standard deviation of the amount of SP (pg/ml) produced in pulp tissue samples

Submucosal injection	SP (pg/mL) (mean±SD)		p-value	Cohen’s d
	Experimental	Control		
Cryotherapy	224.94±42.32	291.24±62.23	0.005*	−1.44
Corticosteroids	297.89±53.17	369.24±83.69	0.049*	−0.84
NSAIDs	343.20±74.89	400.02±54.24	0.005*	−1.42

*: significant (p<0.05); ns: non-significant (p>0.05)

The percentage reduction of SP between the different groups (p=0.015) was significantly different. The greatest percentage reduction in the level of SP was found with the cryotherapy group (1.43±0.33), followed by dexamethasone (1.14±0.28), while the lowest percentage reduction was found with NSAIDs (1.02±0.17). *Post hoc* pairwise comparisons showed cryotherapy to have significantly greater reduction than diclofenac (p<0.001).

### Correlations between the reduction in IL6 and SP

**Figure 3:**
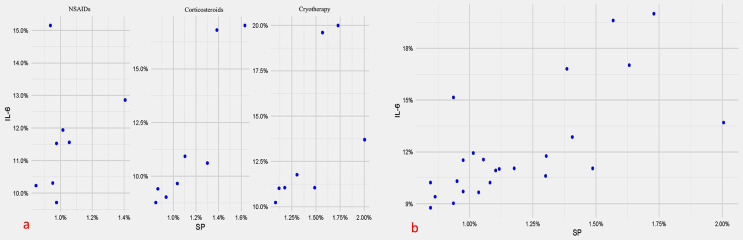
Scatter plot showing the correlations between the change in IL-6 and SP a) within each group; b) overall

Overall and within both cryotherapy and corticosteroids groups, the change in IL6 and SP (rs>0.7, p<0.05) showed a strong positive significant correlation. For the NSAIDs group, the correlation was not statistically significant (p=0.399), as shown in Figure 3 .

## Discussion

Many studies have investigated the effect of different agents on the level of pain perception during or after endodontic treatment such as premedication with NSAIDs,^15^ steroids,^15 , 20^ or opioids.^21^ Recently interest in investigating the effect of these factors at the molecular level has been growing. Several studies have considered the effect of different factors on the level of cytokines expression in the dental pulp tissue such as the effect of different premedications,^11 , 20^ anaesthetic solutions and vasoconstrictors,^22^ extraction methods,^3^ rotary instrumentation systems,^23^ bleaching,^24^ and occlusal trauma^25^ on the level of proinflammatory cytokines released in pulp tissue.

The literature reports that treatment with NSAIDs or corticosteroids can significantly reduce the level of pain perception^15^ and inflammatory cytokines expressed in dental pulp tissue.^11 , 20^ However, the well-known side effects of these drugs, gastrointestinal upset, increased appetite, insomnia, and mood changes, made it inevitable to search for a safer replacement that provides a similar favourable effect.

Several studies reported in the literature have proved a high success rate for the use of cryotherapy as a safe method in reducing pain experience. Cryotherapy was utilized in multiple different ways in endodontics either intracanal cryotherapy via final irrigation with cold saline^26^ to lower the outer root surface temperature,^27^ application of cold packs after endodontic treatment intra-orally or extra-orally^28^ and to control swelling and pain after endodontic surgery.^29^ However, no study was found discussing the effect of cryotherapy by submucosal infiltration injection of cold saline into the tissues surrounding the affected tooth. This study is considered novel in investigating this approach to cryotherapy application.

Pulpal inflammation can be successfully induced in animal models such as mice by intentional exposure of the pulp and subsequent infection from the bacteria of the oral cavity.^30 , 31^ Therefore, induced pulpal exposure in animals was considered a stable and reproducible experimental model that could serve in studying the progress of the inflammatory process in pulp tissue as well as the effect of different factors on this process.

Rabbit models for pulpal inflammation were considered to have the combined advantage of providing an appropriate sample size and a relatively large volume of samples for better and easier pulp tissue collection and detection of the mediators under the study.

This study was consistent with the results reported by He, et al.^19^(2017), in their study on mice where samples were collected after 6 hours showing a peak of inflammatory cytokines level of expression in pulp tissue, which allowed for more accurate evaluation and comparison of the effect of different injection solutions.

In this study, there was a significant reduction in the level of IL6 and SP released in pulp tissue subjected to injection by cold saline (cryotherapy), dexamethasone sodium phosphate, and diclofenac sodium compared with control samples where no injection was performed. This is a quite logical and expected finding although not previously tested. Diclofenac sodium and dexamethasone sodium phosphate are well-known anti-inflammatory drugs that blocks the inflammatory cascade by cyclooxygenase and phospholipase inhibition, respectively. Cryotherapy eliminates heat from tissues thus decreasing their temperature.^32^ This reduction in tissue temperature results in vasoconstriction which limits tissue edema^33^, as well as restricting the level of cell metabolism resulting in decreasing oxygen consumption, thereby reducing the release of free radicals locally in tissues which reduces the level of tissue damage.^32 , 33^ The level of inflammatory enzymes also increases with an increase in tissue temperature.^34^ Cold application locally on damaged skin has been found to induce alteration of pain threshold and results in pain reduction.^35^ It also affects the nerve conduction capacity of nociceptors as well as thermoreceptors, which are temperature-sensitive pain receptors, stimulated by variation in tissue temperature. Cryotherapy can induce activation of these thermoreceptors thus blocking nociception and pain perception.^32^

Results of the current study are consistent with those reported by Nguyen, et al.^20^ (2020) who used systemic preoperative ibuprofen and found a reduction in the level of IL6 expression. They could also explain the results reported by Gorecki, et al.^36^(2018), Aksoy, et al.^21^(2020), and Yavari, et al.^37^ (2019) in the randomized control trials that revealed a significant reduction in postoperative pain experience in cases undergoing preoperative submucosal injection of diclofenac and dexamethasone respectively. Keskin, et al.^38^ (2017) reported a reduction in the level of IL6 released in periapical exudate as well as the level of postoperative pain in cases treated with intracanal cryotherapy.

Although no studies evaluated the effect of submucosal injection of cold saline (cryotherapy), steroids, or diclofenac sodium on the SP level expressed in pulp tissue, Abbate, et al.^39^ (2016) demonstrated no significant reduction in the SP expression level in experimentally exposed pulp tissue with preoperative systemic ketoprofen ingestion. This difference could be due to the short time (15 minutes) allowed for the release of cytokines in their study before sample collection and analysis.

Dexamethasone sodium phosphate resulted in a more statistically significant reduction in SP pulpal release than diclofenac sodium. This could be explained based on the mechanism of action of both drugs since dexamethasone blocks the phospholipase enzyme blocking the inflammatory cascade at a higher level than the semi-selective cyclooxygenase-2 inhibition done by the diclofenac sodium.

On the other hand, diclofenac sodium reduced the pulpal release of IL6 more than dexamethasone in a statistically significant way. This is an unexpected and yet unexplained finding. Comparison to the literature is inapplicable since no study has compared these two classes of drugs on pulpal release of IL6.

Cryotherapy reduced the pulpal release of both SP and IL6 more than the other two tested drugs. An explanation for this finding is the submucosal route of delivery of the cryotherapy which affects the vascularity in the area of interest performing the aforementioned mechanisms locally. Again, no direct comparison of our results to the literature is applicable since this is the first evaluation of the submucosal injection of cold saline (cryotherapy) on the pulpal release of SP and IL6. However, this results could serve as evidence that submucosal cryotherapy can be adopted as an efficient, safer and cost-effective replacement of submucosal injection with anti-inflammatory drugs for controlling the inflammatory process and the release of pain mediators and thus potentially reducing the pain experience.

This study revealed a strong and positive correlation between SP and IL6 reduction in all tested injections. Although SP has been shown to stimulate the production of IL6 in immune cells like mast cells, macrophages, and monocytes, the exact mechanisms and implications of this interaction may require further research.

Despite similarities, the genetic, physiological, and anatomical differences between animals and humans are significant. What works in an animal model may not necessarily work the same way in humans. Many human diseases are multifaceted and influenced by genetic, environmental, and lifestyle factors. Animal models may oversimplify these complexities, leading to findings that do not fully translate to human conditions. While animal models can provide insights into behavior and cognition, there are inherent differences in cognitive processes and social behaviors between species. This makes it challenging to directly translate findings from animal behavioral studies to human behavior.

Yet, many basic biological processes are conserved across species. This means that fundamental physiological and biochemical mechanisms in animals can often be relevant to humans. Animal studies are frequently used to assess the safety and efficacy of new drugs and routes of administration before human trials. If a drug shows promising results in animals, the likelihood of it having similar effects in humans is higher. Animals can be genetically modified or bred to mimic certain human diseases, allowing researchers to study the underlying mechanisms and test potential treatments. These models can sometimes accurately reflect aspects of human diseases.

Given these correlations and limitations, careful interpretation of findings from animal studies and consideration within the broader context of human biology and disease are deemed mandatory. Additionally, integrating multiple lines of evidence from various research approaches, including animal studies, *in vitro* studies, epidemiological data, and clinical trials, can provide a more comprehensive understanding of complex biological processes and diseases.

Despite the limitations of this study, being on an animal model and testing only SP and IL6, these results are quite promising. Further investigation of submucosal cryotherapy as an alternative to premedication on the effect of inferior alveolar nerve block in cases of hot mandibular molars is deemed mandatory.

## Conclusion

Submucosal cryotherapy reduces the pulpal release of SP and IL6 and could be tested as an alternative to premedication to potentiate the effect of anesthesia and control postoperative endodontic pain.
